# Maltol ameliorates intervertebral disc degeneration through inhibiting PI3K/AKT/NF-κB pathway and regulating NLRP3 inflammasome-mediated pyroptosis

**DOI:** 10.1007/s10787-022-01098-5

**Published:** 2022-11-19

**Authors:** Yuhang Gong, Jianxing Qiu, Ting Jiang, Ze Li, Weikang Zhang, Xiaohang Zheng, Zixuan He, Weifu Chen, Zhangfu Wang, Xingbing Feng, Meizhen Wang, Zhenghua Hong

**Affiliations:** 1 Department of Orthopaedics, Taizhou Hospital Affiliated to Wenzhou Medical University, Linhai, 317000 Zhejiang Province People’s Republic of China; 2 Bone Development and Metabolism Research Center, Taizhou Hospital of Zhejiang Province, Linhai, 317000 Zhejiang Province People’s Republic of China; 3 Department of Orthopaedics, Taizhou Hospital of Zhejiang Province, Zhejiang University, Linhai, 317000 Zhejiang Province People’s Republic of China; 4 Department of Operation Room, Taizhou Hospital Affiliated to Wenzhou Medical University, Linhai, 317000 Zhejiang Province People’s Republic of China

**Keywords:** Intervertebral disc degeneration, Maltol, ECM, PI3K/AKT, NK-κB, Pyroptosis

## Abstract

**Objectives:**

As one of the major causes of low back pain, intervertebral disc degeneration (IDD) has caused a huge problem for humans. Increasing evidence indicates that NLRP3 inflammasome-mediated pyroptosis of NP cells displays an important role in the progression of IDD. Maltol (MA) is a flavoring agent extracted from red ginseng. Due to its anti-inflammatory and antioxidant effects, MA has been widely considered by researchers. Therefore, we hypothesized that MA may be a potential IVD protective agent by regulating NP cells and their surrounding microenvironment.

**Methods:**

In vitro, qRT-PCR, and Western blot were used to explore the effect of MA on the transcription and protein expression of the anabolic protein (ADAMTS5, MMP3, MMP9) catabolic protein (Aggrecan), and pro-inflammatory factor (iNOS COX-2). Next, the effects of MA on PI3K/AKT/NF-κB pathway and pyroptosis pathway were analyzed by Western blot and immunofluorescence. Molecular docking was used to investigate the relationship between PI3K and MA. Moreover, ELISA was also used to detect the effects of MA on inflammatory factors (TNF-α, PGE2, IL-1β, and IL-18). In vivo, the effects of MA on the vertebral structure of IDD mice were studied by HE and SO staining and the effects of MA on ECM and PI3K/AKT/NF-κB and pyroptosis pathway of IDD mice were studied by immunohistochemical staining.

**Results:**

MA can ameliorate intervertebral disc degeneration in vivo and in vitro. Specifically, the molecular docking results showed that the binding degree of MA and PI3K was significant. Second, in vitro studies showed that MA inhibited the degradation of ECM and inflammatory response by inhibiting the PI3K/AKT/NF-κB pathway and the pyroptosis mediated by NLRP3 inflammasome, which increased the expression of anabolic proteins, decreased the expression of catabolic proteins, and decreased the secretion of inflammatory mediators such as IL-18 and IL-1β. In addition, according to the study results of the mouse lumbar instability model, MA also improved the tissue disorder and degradation of the intervertebral disc, reduced the loss of proteoglycan and glycosaminoglycan, and inhibited intervertebral disc inflammation, indicating that MA has a protective effect on the intervertebral disc to intervertebral disc in mice.

**Conclusions:**

Our results suggest that MA slowed IDD development through the PI3K/AKT/NF-κB signaling pathway and NLRP3 inflammasome-mediated pyroptosis, indicating that MA appeared to be a viable medication for IDD treatment.

## Introduction

Research studies have reported that low back pain (LBP) has become a problem for a large proportion of the global population (Dereje 2020), increasing patients’ medical costs and reducing their quality of life (Andersson 2018). Moreover, intervertebral disc degeneration (IDD) is one of the leading causes of LBP (Smith et al. 2011, Katharina Trompeter et al. 2017), but there is no wonderful curative drug available to address this condition (Hickman et al. 2022). The main structures of the intervertebral disc (Humzah et al. 1988) are the innermost nucleus pulposus (NP), the annulus fibrosus, which is wrapped around the outer part of the NP, and the endplates, which are in contact with the upper and lower vertebral bodies (Cassidy et al. 1989). The normal NP is a gel-like structure that allows the disc to withstand mechanical stresses from resting and moving bodies (Ming-Liang et al. 2018; Kadow et al. 2015), and it is now generally accepted that degeneration of the normal NP is the first step in causing IDD (Ding et al. 2013; Buckwalter 1995). The extracellular matrix (ECM) secreted by NP cells (NPCs) is a key area of pressure absorption by the NP tissue (Sakai et al. 2015), which also suggests that NPCs are critical for disc formation. Normal NPCs maintain the structural stability of the ECM by secreting anabolic matrix proteins such as Aggregan and collagen II (Le Maitre et al. 2007), but when NPCs become abnormal, the ECM is disrupted and becomes anabolic deficient and excessively catabolic through the secretion of ADAMTS family proteins and MMP family proteins and the increased secretion of inflammation family proteins as well as insufficient secretion of Aggregan and Collagen II, which further leads to more serious IDD (Roughley et al. 2004; Liu et al. 1991).

Interleukin-1β (IL-1β) is the most significant cytokine in the IL-1β family and has a vital role in IDD evolution and progression (Wang et al. 2020), and it is an extremely pleiotropic cytokine. In general, IL-1β forms a complex with the Toll-like/IL-1 receptor (TIR) domain (Martin et al. 2018) and then interacts with myeloid differentiation factor 88 (MyD88), which activates the downstream signaling pathways, such as PI3K/AKT pathway (Yang et al. 2015). This process induces an inflammatory response and the secretion of a number of inflammatory factors, such as IL-6, tumor necrosis factor-α (TNF-α), inducible nitric oxide synthase 2 (iNOS2), and cyclooxygenase-2 (COX-2). It can alter ECM metabolism (Risbud et al. 2014) and accelerate the process of IDD. Therefore, IL-1β was used as an in vitro stimulus in the present study. Inflammation of the intervertebral disc is a key cause of IDD (Peng et al. 2015). Earlier research has shown that the nuclear factor kappa B (NF-κB) signaling pathway (Wuertz et al. 2012) is critical for regulating the inflammatory response and ECM catabolism, as well as in IDD formation in mice (Zhao et al. 2015). Phosphorylation and nuclear translocation of the NF-κB p65 subunit (p65) to the nucleus stimulate transcription of the specific gene and is frequently associated with a nuclear localization signal on the phosphorylated IκBα-exposed NF-κB complex (Jia et al. 2020), and the inflammatory factor IL-1β can induce the occurrence of this manifestation (Hayden et al. 2004).

The phosphoinositide 3-kinase (PI3K)/AKT pathway signaling pathway (Cantley et al. 2002) is a member of the serine/threonine kinase-related signaling family that can regulate the NF-κB signaling pathway by phosphorylating p65 and IκBα. This might also imply that inflammatory factors activate both the PI3K/AKT and NF-κB signaling pathways (Datler et al. 2019; Tarassishin et al. 2011). Furthermore, the findings revealed that the NF-κB signaling pathway is downstream of the PI3K/AKT signaling pathway during inflammation (Xiao et al. 2022; Ni et al. 2022). Consequently, PI3K is a potential candidate for IDD therapy, and the PI3K/AKT/NF-κB signaling pathway is one of the major signaling routes for IDD treatment.

The NLR family pyrin domain containing 3 (NLRP3) inflammasomes has been associated with the advancement of several diseases (Zhang et al. 2020; Zhou et al. 2011). Caspase-1 was typically activated by the NLRP3 inflammasome complex. The activated caspase-1 was further cleaved into cleavage caspase-1 (cleaved-caspase-1) and induced cell cleavage and pyroptosis to promote the massive release of the inflammatory factors IL-1β and IL-18 (Yansong et al. 2019; Khan et al. 2018). NLRP3 inflammasomes have been linked to IDD in earlier research as a target gene for p65 phosphorylation (Chen et al. 2020). Furthermore, the NLRP3 inflammasome may facilitate the expression of inflammatory factors in NPCs, thereby accelerating the course of IDD (Chao-Yang et al. 2021; Chen et al. 2015).

Maltol (MA) (3-hydroxy-2-methyl-4-pyrone), obtained by steaming the traditional Chinese medicine red ginseng, is an aromatic compound (Lee, et al. 2000). Previous studies have shown that MA has a significant role in inflammation inhibition, antioxidant and anti-aging (Xing, et al. 2022; Sha, et al. 2021). It was found that MA protected the liver from the acute attack on CCl_4_ by inhibiting apoptosis and inflammation-induced injury (Liu, et al. 2018). In addition, MA reduces the inflammatory responses of osteoarthritis through the PI3K/AKT/NF-κB pathway (Lu, et al. 2021). However, the effects and potential mechanisms of MA on NPCs remain unknown. Therefore, based on the anti-inflammatory and osteoprotective effects, the relationship between the anti-inflammatory actions of MA in IDD and the PI3K/AKT/NF-κB signaling pathway and NLRP3-mediated pyroptosis requires further validation. We hypothesized that MA slowed the course of IDD by inhibiting cell death via the PI3K/AKT/NF-κB and pyroptosis signaling pathway. Our research was intended to provide greater evidence for the use of MA in IDD treatment.

## Materials and methods

### Reagents and antibodies

Maltol (purity ≥ 95.0%) and 740YPDGFR (purity > 99%) were purchased from MCE (Shanghai, China). Dimethyl sulfoxide (DMSO) was from Sigma-Aldrich (St. Louis, USA). Recombinant IL-1β was purchased from Novoprotein (Suzhou, China). MMP-3 (Cat# ab76110), MMP-9 (Cat# ab124995), ADAMTS-4 (Cat# ab179475), and ADAMTS-5 (Cat# ab19027) were from Abcam (Cambridge, UK). p-IκBα (Cat# 5209), IκBα (Cat# 4814), p-p65 (Cat# 3033), p65 (Cat# 8242 T), PI3K (Cat# 17366), p-AKT (Cat# 4060), AKT (Cat#C67E7), and β-actin (Cat# 3700) were from cell signaling technology (Beverly, MA, USA). p-PI3K (Cat# ab179475) was purchased from Immunoway (Plano, TX, USA). iNOS (Cat# A3774) and IL-1β (Cat# A19635) were from Abclonal (Wuhan, China). Aggrecan (Cat# PA1-1746) was purchased from Invitrogen (Waltham, MA, USA). Collagen II (Cat# K009364P) was purchased from Solarbio (Beijing, China). COX2 (Cat# EM1902-12), Alexa Fluor®488 (Cat# HA1104), and Alexa Fluor®594 (Cat# HA1105) were purchased from Huabio (Hangzhou, China). Goat anti-rabbit IgG (H + L) HRP (Cat# HA1104 S0001) was obtained from Affbiotech (Guangzhou, China). 4′,6-diamino-2-phenylindole (DAPI) was purchased from Biyuntian (Shanghai, China). Cell culture reagents were obtained from Lanso (Jiaxing, China).

### Acquisition and culture of rat NPC lines

The NPCs line was donated by Prof. Dee Chen, Rush University, Chicago, IL, USA. Myeloid cells were incubated with 10% fetal bovine serum (VWR, Melbourne, Australia) and 1% antibiotic (streptomycin/penicillin) in Dulbecco's Modified Eagle Media: Nutrient Mixture F-12 (DMEM/F12) at 37 °C under 5% CO2 in an incubator. The complete medium (containing 10% FBS and 1% antibiotic in DMEM/F12) was replaced every other day, and the culture condition (at 37 °C under 5% CO2 in an incubator) remained the same after each replacement. The DMSO concentration in all experiments was less than 1:1,000. When cells attained 80–90% confluence, they were trypsinized with 0.25% trypsin–EDTA, passaged, and transplanted to 10-cm culture plates at the appropriate density (300,000 cells/well).

### Experimental design

For in vitro experiments, NPCs were pretreated with IL-1β (10 ng/mL) for 1 day and subsequently treated with additional or different MA concentrations (5, 10, 20 μM) alone, with no treatment in the control group. Cells were collected after 24 h of incubation for further experiments. For in vivo experiments, mice were randomly assigned to four groups (control group, spinal instability group, spinal instability + 15 mg/kg MA group, and spinal instability + 30 mg/kg MA group). The mice in the spinal instability group and MA groups were subjected to surgical manipulation to generate the lumbar spine instability model. Following surgery, the corresponding drug treatment was administered according to the grouping. Mice in the control group were treated with phosphate-buffered saline (PBS), while those in the MA groups received 15 mg/kg or 30 mg/kg MA (dissolved in PBS) by gavage twice a week, respectively, and the IDD group was not treated with the drug. Subsequently, mice were euthanized 12 weeks after surgery with excessive 1% pentobarbital, and IVD tissues were taken for histological and immunohistochemical analysis.

### Cell viability assay

Cell viability was determined using the counting kit-8 (CCK-8 kit) as directed by the manufacturer. In brief, NPCs were incubated in 96-well plates (3000 cells/well) for 24 h before MA treatment. The medium was aspirated after 24, 48, and 72 h of treatment, and 100 μL DMEM/F12 containing 10 μL CCK-8 solution was added and incubated for 1 h at 37 °C in the dark. The absorbance of the wells was determined at 450 nm by Enzyme standard instrument (Thermo Fisher, Waltham, MA, USA).

### Quantitative real-time polymerase chain reaction (qRT-PCR)

Total RNA was isolated from treated NPCs using the Trizol method. The PrimeScript-RT kit (Cwbio, Taizhou, China) and the Real-Time PCR equipment (Bio-Rad, CA, USA) were used to synthesize cDNA from 1 µg total RNA. The ABI 7500 real-time PCR machine (Applied, CA, USA) and SYBR Premix Ex Taq (Vazyme, Nanjing, China) were used to amplify cDNA. Cycling threshold (Ct) values were calculated and normalized to the level of a housekeeping gene (GAPDH). The 2-ΔΔCt technique was used to determine the expression of target genes in various groups. The primers of the rat used are presented in Table 1.

**Table 1:** Specific primers used in quantitative real-time PCR (qRT-PCR)

Number	Target gene	Forward (5’–3’)	Reverse (5’–3’)
1	*ADAMTS-4*	CGC​T GA​GT A​GAT ​TCG​T GG​AG A​C	AGT​T GA​CA G​GG T​TTC​ GGA​T GC
3	*ADAMTS-5*	CGA​C AA​GA G​TCT​ GGA​G GT​GA G	CGT​G AG​CC A​CAG​ TGA​A AG​C
5	*Aggrecan*	GAC​C AG​GA G​CAA​ TGT​G AG​GA G	CTCG CGGT CGGG AAAG T
7	*COX-2*	TGAT CTAC CCTC CCCA CGTC	ACAC ACTC TGTT GTGC TCCC
8	*GAPDH*	ATG​G GA​AG C​TGG ​TCA​T CA​AC	GTG​G TT​CA C​ACC​ CAT​C AC​AA
10	*iNOS*	GAGC AAAA AAGG GCAA CAC	CGCA CTTC TGTC TCTC CAAA
11	*MMP-3*	GAC​C AG​GG A​CCA​ ATG​G AG​AT G	TGA​GC A​GCA​ ACC​A GG​AA T​AGG
12	*MMP-9*	CCT​A CT​GC T​GGT​ CCT​T CT​G	GTG​G TT​CA C​ACC​ CAT​C AC​AA

### Western blot analysis

Following appropriate processing, NPCs were lysed on ice with radio immunoprecipitation assay (RIPA) buffer and phenylmethylsulfonyl fluoride (PMSF) and centrifuged at 12,000 rpm and 4 °C for 15 min. Protein concentrations were determined using the BCA Protein Assay Kit (Biosharp, Anhui, China) according to the manufacturer's instructions. A total of 40 μg protein was separated by 8–12% SDS–PAGE and blotted onto polyvinylidene fluoride membranes (Millipore, CA, USA). Following blocking with protein blocking solution for 1 h, the membranes were incubated with MMP-3 (1:1000), MMP-9 (1:1000), ADAMTS-4 (1:1000), ADAMTS-5 (1:1000), Caspase1 (1:1000), NLRP3 (1:1000), p-IκBα (1:1000), IκBα (1:1000), p-p65 (1:1000), p65 (1:1000), PI3K (1:1000), p-AKT (1:1000), AKT (1:1000), p-PI3K (1:1000), iNOS (1:1000), IL-1β (1:1000), Aggrecan (1:1000), COX2 (1:1000), iNOS (1:1000),and β-actin (1:5,000) primary antibodies overnight at 4 °C, followed by incubation with the corresponding secondary antibodies for 1 h at room temperature. The membranes were washed three times with Tris Buffered Brine and Tween20 mixture (TBST) and observed with chemiluminescence plus reagent (Millipore, Burlington, MA, USA). The signals were visualized using ImageQuant LAS 500 (GE Health Care, CA, USA) and the images were quantified using ImageJ software (BioRad, CA, USA).

### Enzyme-linked immunosorbent assay (ELISA)

The PGE2 (Cat: AE90706Ra, AMEKO, China), TNF-α (Cat: AE90301Ra AMEKO, China), IL-1β (Cat: AE90731Ra AMEKO, Jiaxing, China), and IL-18 (Cat: AE90232Ra AMEKO, Jiaxing, China) levels in the cell supernatant collected after treatment were determined using the appropriate ELISA kits according to the manufacturer's instructions.

### Immunofluorescence

Following the NPCs cultivation, the entire medium was withdrawn and the cells were washed three times with PBS before being treated with 4% paraformaldehyde (PFA) for 15 min, dissolved in 0.3% Triton X-100 in PBS for 10 min, and blocked with5% bovine serum albumin (BSA) at 37 °C for 1 h. The cells were incubated with the respective primary antibodies (1:200) for NLRP3, Collagen II, and p65 overnight at 4 °C. Subsequently, the cells were incubated for 1 h at room temperature with Alexa Fluor®488 or Alexa Fluor®594. Finally, the anti-fluorescence quencher containing DAPI was applied and incubated for 5 min at 37 °C. On completion of the above methodology, the microscope (Nikon, Tokyo, Japan) was used to randomly select a cell field for observation and images obtained and stored. ImageJ was used to calculate the fluorescence intensity.

### Molecular docking

The molecular structure of MA was obtained from the PubChem database, and the PI3K protein structure imported into the database was obtained from the RCSB protein database (https://www.rcsb.org/). AutoDock software (http://vina.scripps.edu/) was used to prepare the ligand and protein required for molecular docking. For the PI3K protein, its crystal structure should be pretreated, including water molecule removal, hydrogenation, amino acid modification, energy optimization, and field parameter adjustment. Then, vina inside PyRx software was used for virtual screening, and its Binding Affinity (Kcal/mol) value represented the Binding ability of the two. The lower the Binding ability, the more stable the ligand and receptor Binding. Finally, DS SolidWorks software (Dassault Systemes, MA, USA) was used for mapping.

### Surgical procedure

The Animal Welfare and Ethics Committee of Taizhou Hospital, Wenzhou Medical University authorized this in vivo animal study, and routine animal care and animal experiments were performed following the National Institutes of Health Guide for the Care and Use of Laboratory Animals. A total of 24 mice were randomly assigned to four groups (six mice per group), control group, IDD group, 15 mg/kg MA group, and 30 mg/kg MA group). The two MA groups and the IDD group were operated at the same time, as described below. The hair on the backs of the mice was removed after general anesthesia with 3% isoflurane to thoroughly expose the surgical region. Subsequently, the skin was disinfected with iodine swabs and cut open. The paraspinous muscle was bluntly split to completely expose the L2–L6 spinous process. The L3–L5 spinous process was excised according to the anatomical landmarks. Following strict clean hemostasis, the incision was closed with sutures. The subsequent daily treatment has been described previously. At 12 weeks postoperatively, the mice were euthanized and the lumbar spine tissue was collected and fixed with 4% PFA. Once specimen decalcification was completed, they were subjected to histological studies.

### Histopathological analysis

The specimens were dehydrated and paraffin-embedded. Subsequently, the tissue was cut into 5 µm sections. HE and SO staining were used to stain each intervertebral disc slide. Experienced histology researchers were asked to use a microscope (Olympus Inc. Tokyo, Japan) to evaluate the cytoarchitecture and morphology of the discs blindly.

### Immunohistochemical analysis

Following dewaxing, the immunohistochemical sections were incubated with % H2O2 for 10 min and rinsed three times with PBS. The slices were incubated with 0.1% trypsin for 20 min at 37 °C before being washed three times with PBS. Sections were blocked with 5% goat serum albumin for 1 h at 37 °C and incubated with primary antibodies (Collagen II, MMP-9, 1:100, NLRP3, p-p65, and p-PI3K, 1:50) overnight at 4 °C. Nonspecific IgG was used to incubate the negative control sections. The sections were rinsed three times with PBS and incubated for 1 h at 37 °C with the HRP-coupled secondary antibody. Each specimen was examined in a minimum of three different regions.

### Statistical analysis

The mean ± standard deviation of separate experiments is used to express the data. GraphPad Prism (La Jolla, CA, USA) was used to analyze the data. For intergroup comparison, we used ANOVA (analysis of variance) with Tukey’s post hoc test. Differences in *p* < 0.05 were considered to be statistically significant.

## Results

### Effects of varying Maltol concentrations on NPCs

**Fig. 1:**
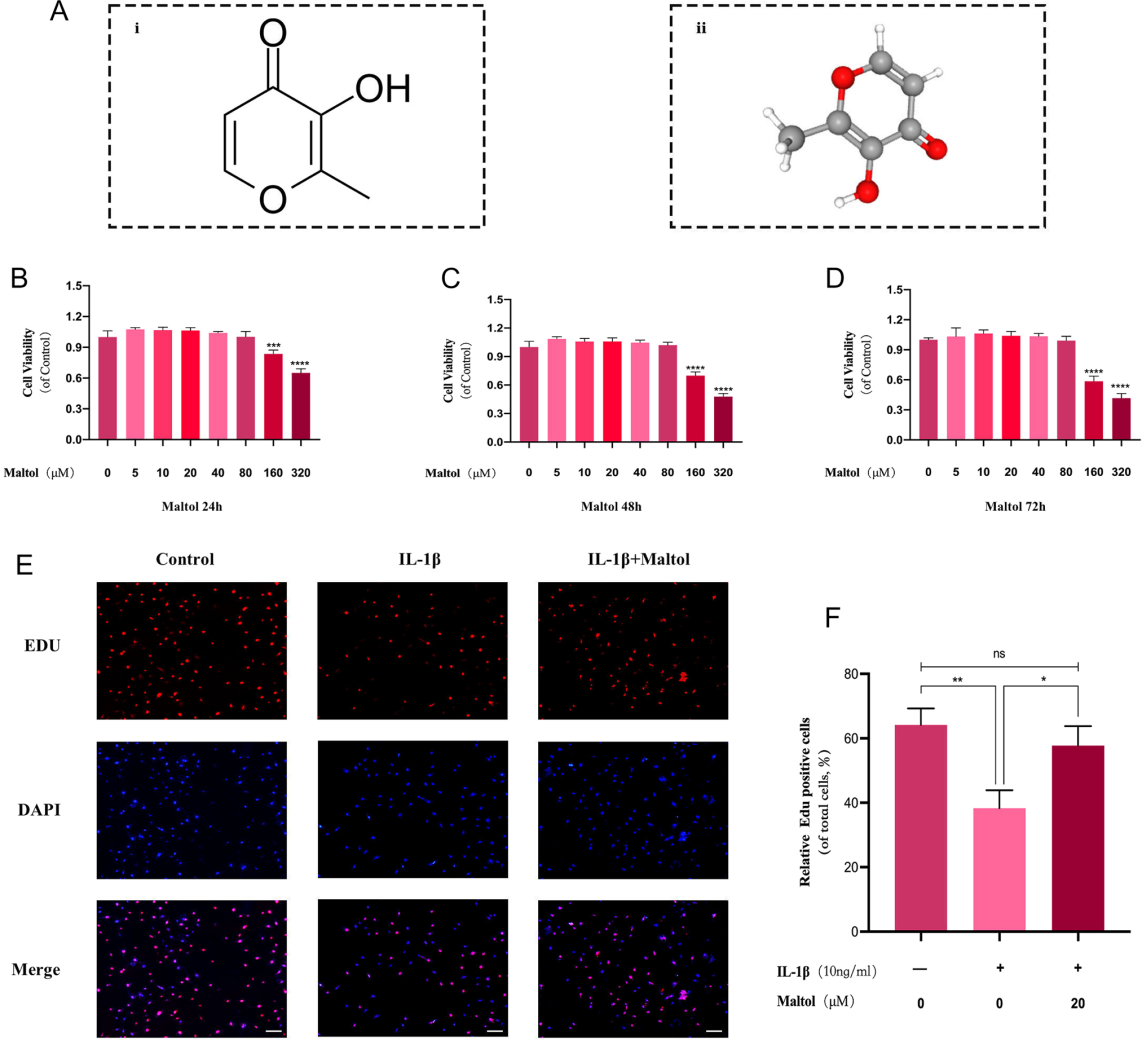
Effect of Maltol on the survival of NP cells. **A** Chemical structure of Maltol was obtained by collecting in station PubChem (https://pubchem.ncbi.nlm.nih.gov). **B**–**D** Cytotoxic effect of Maltol (0, 5, 10, 20, 40, 80, 160, 320 µM) in NP cells was determined at various concentrations for 24 h, 48 h and 72 h using a CCK8 assay. **E** EdU staining assay was detected the effect of Maltol (20 µM) on the proliferation of NP cells treated by IL-1β (10 ng/mL) (scale bar: 100 µm). **F** Relative Edu positive cells of total cells (%) was determined using ImageJ. The values presented are the means ± SD of three independent experiments. ns *p* > 0.05, *p < 0.05, **p < 0.01, ***p < 0.001, ****p < 0.001

The chemical structure of Maltol (MA) is shown in Fig. 1A. To determine whether MA has cytotoxic effects on NPCs, we treated these cells for 24, 48, and 72 h (Fig. 1B–D) with different MA concentrations (0, 5, 10, 20, 40, 80, 160, 320 μM) and examined them using the CCK-8 kit. After 72 h of treatment, MA doses up to 80 μM were not substantially cytotoxic to NPCs (Fig. 1B). Subsequently, NPCs were pretreated with IL-1β followed by (0, 5, 10, and 20 µM) MA. According to EdU staining results, the proliferation of NPCs was suppressed by IL-1β, while MA was able to dramatically promote cell proliferation among IL-1β-treated NPCs (Fig. 1E), and MA could significantly revert the cell proliferation (Fig. 1F). The results showed that MA had no toxic effect on NPCs at concentrations below 20 μM and promoted the proliferation of nucleus pulposus cells.

### Effects of MA on the ECM in IL-1β-treated NPCs

**Fig. 2:**
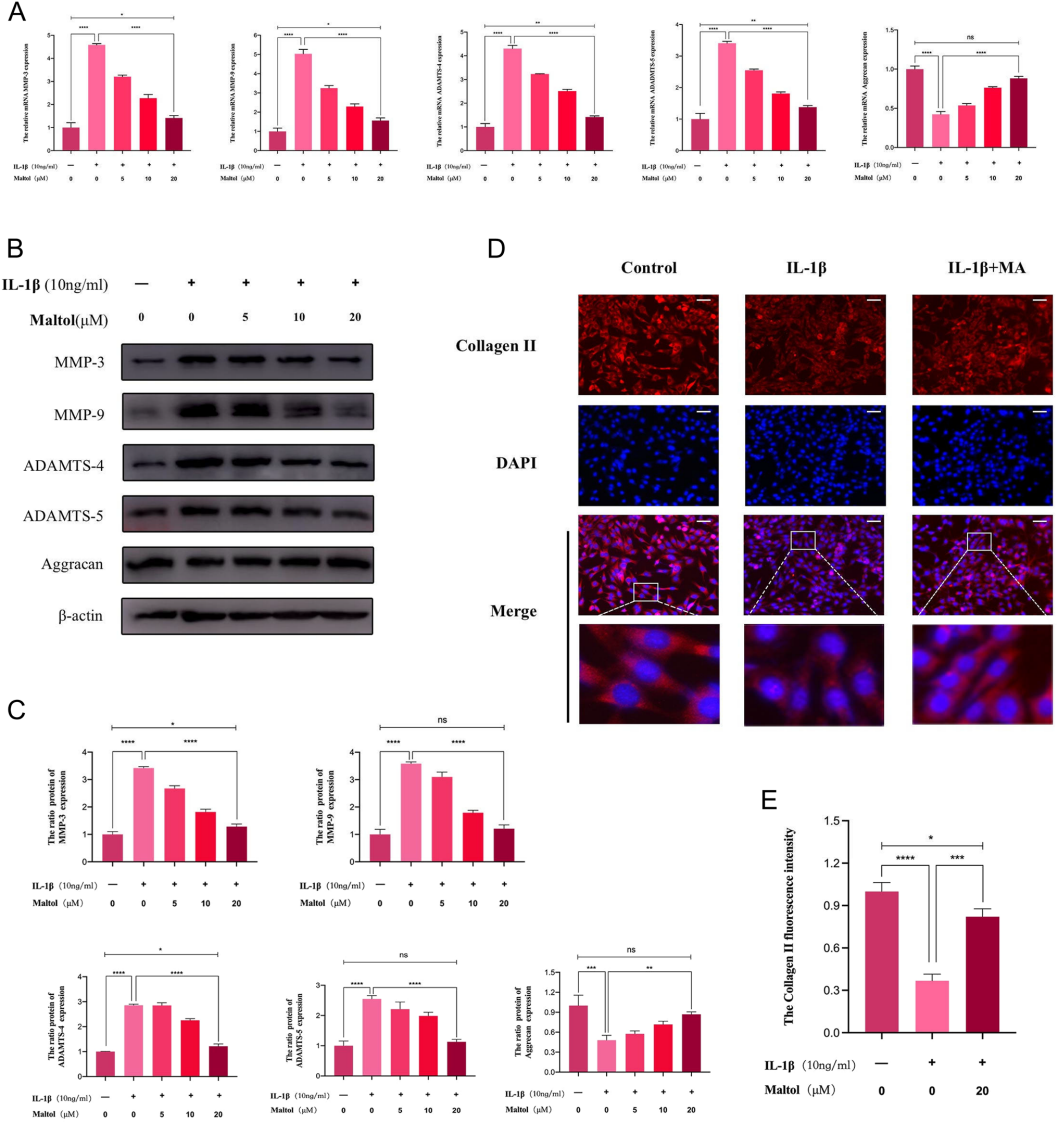
Effects of Maltol on the ECM degradation in IL-lβ-treated NP cells. **A** Gene expression of the anabolic and catabolic proteins, such as MMP-3, MMP-9, ADAMTS-4, ADAMTS-5, and Aggrecan were detected by qPCR in the NP cells, treated with or without the administration of Maltol with IL-lβ for 24 h. **B** Protein expression of ADAMTS-4, ADAMTS-5, MMP-3, MMP-9, Aggracan was detected by Western blot in NP cells, treated with or without administration of Maltol with IL-lβ for 24 h. **C** Levels were determined using ImageJ. **D** Collagen II was detected by immunofluorescence combined with DAPI staining for nuclei (scale bar: 100 µm). **E** Fluorescence intensity of Collagen II was determined using ImageJ. The values presented are the means ± SD of three independent experiments. ns *p* > 0.05, *p < 0.05, **p < 0.01, ***p < 0.001, ****p < 0.0001

We analyzed the effects of MA on the anabolism and catabolism of the ECM. The qRT-PCR results showed that catabolic markers ADAMTS-4, ADAMTS-5, MMP-3, and MMP-9 in the IL-1β-treated group were significantly higher than that in the control group (Fig. 2A). By contrast, these catabolic markers were decreased to some extent under MA treatment. In contrast, the anabolic markers collagen II and aggrecan were elevated. Western blot results confirmed that there was a significant decrease in the ratio of aggrecan/β-actin was significantly decreased in NPCs after IL-1β stimulation, at the same time, there was a significant increase in the ratio of ADAMTS-4/β-actin, ADAMTS-5/β-actin, MMP-3/β-actin, and MMP-9/β-actin (Fig. 2B, C). Consistent with the results of qRT-PCR results, the ratio of aggrecan/beta-actin levels recovered after MA treatment, and there was a significant decrease in the ratio of ADAMTS-4/β-actin, ADAMTS-5/β-actin, MMP-3/β-actin, MMP-9/β-actin (Fig. 2B, C). Immunofluorescence results showed that the Collagen II level was decreased after IL-1β treatment compared to the control group, which was reversed by MA (Fig. 2D, E).

### Effects of MA on the expression of inflammatory factors in IL-1β-treated NPCs

**Fig. 3:**
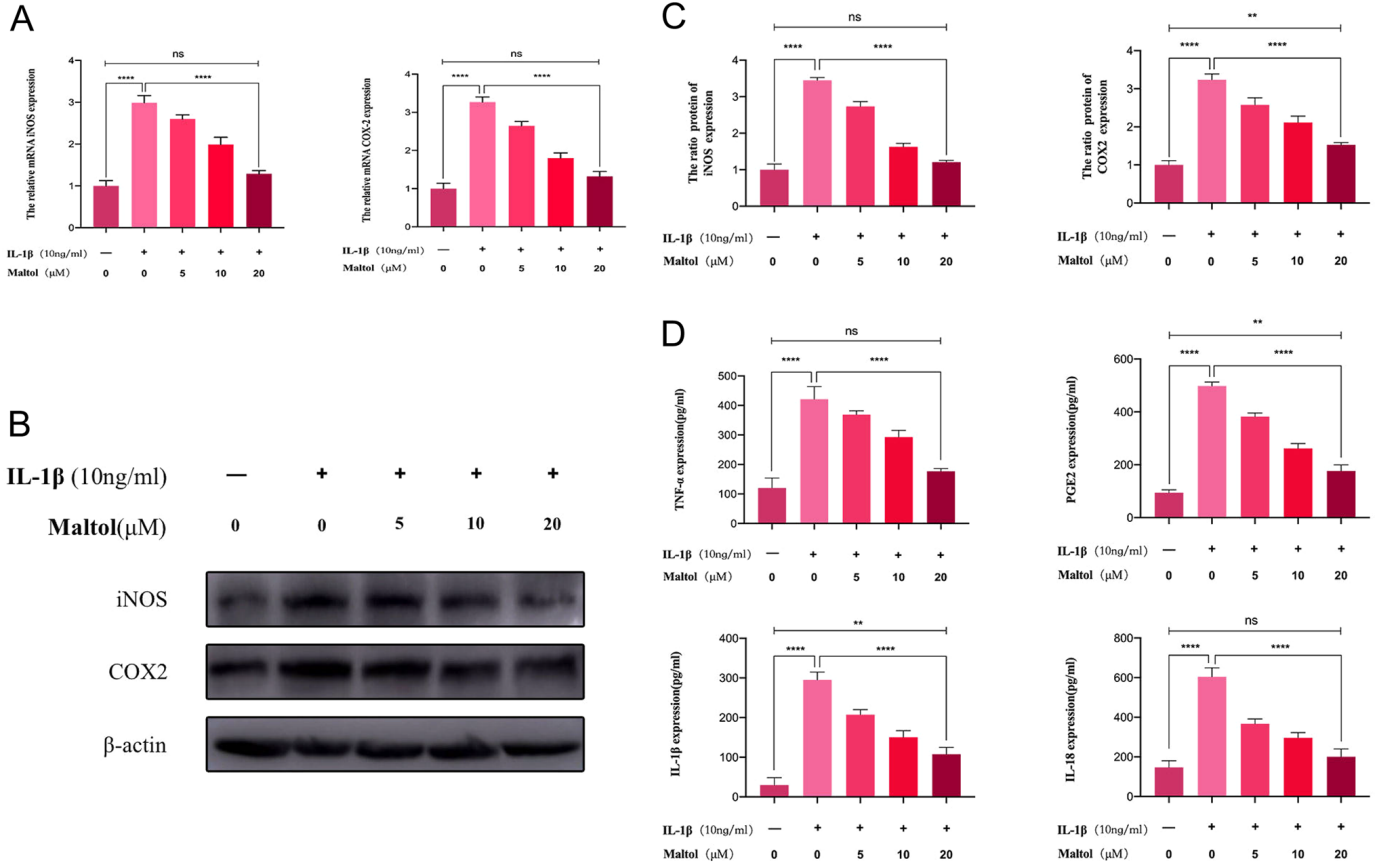
Effects of Maltol on inflammation factors in IL-1β-treated NP cells. **A** mRNA expression of iNOS and COX-2 was detected by qRT-PCR in NP cells, treated with or without administration of Maltol with IL-1β for 24 h. **B** Protein expression of iNOS and COX-2 was detected by Western blot in NP cells, treated with or without administration of Maltol with IL-1β for 24 h. **C** Levels were determined using ImageJ. **D** Expression of TNF-a, PGE2, IL-1β and IL-18 in NP cells was analyzed by ELISA. The values presented are the means ± SD of three independent experiments. ns *p* > 0.05, *p < 0.05, **p < 0.01, ***p < 0.001, ****p < 0.0001

Subsequently, the qRT-PCR, Western blot, and ELISA were applied to investigate whether MA decreased the synthesis of pro-inflammatory comportments at the mRNA and protein levels. The qRT-PCR showed that IL-1β promoted the transcription of iNOS, and COX-2, while MA inhibited them (Fig. 3A). Western blot showed that after IL-1β stimulation, the ratios of iNOS/β-actin and COX-2/β-actin were significantly elevated. When ma-treated NPCs were compared with IL-1β group, the ratios of iNOS/β-actin and COX-2/β-actin were significantly decreased in ma-treated NPCs (Fig. 3B, C). Furthermore, TNF-α, PGE-2, IL-1β, and IL-18 expression were all increased in the presence of IL-1β according to the ELISA results, whereas MA reversed the TNF-α, PGE-2, IL-1β, and IL-18 expression (Fig. 3D).

### Effects of MA on NLRP3 inflammasome-mediated pyroptosis in IL-1β-treated NPCs

**Fig. 4:**
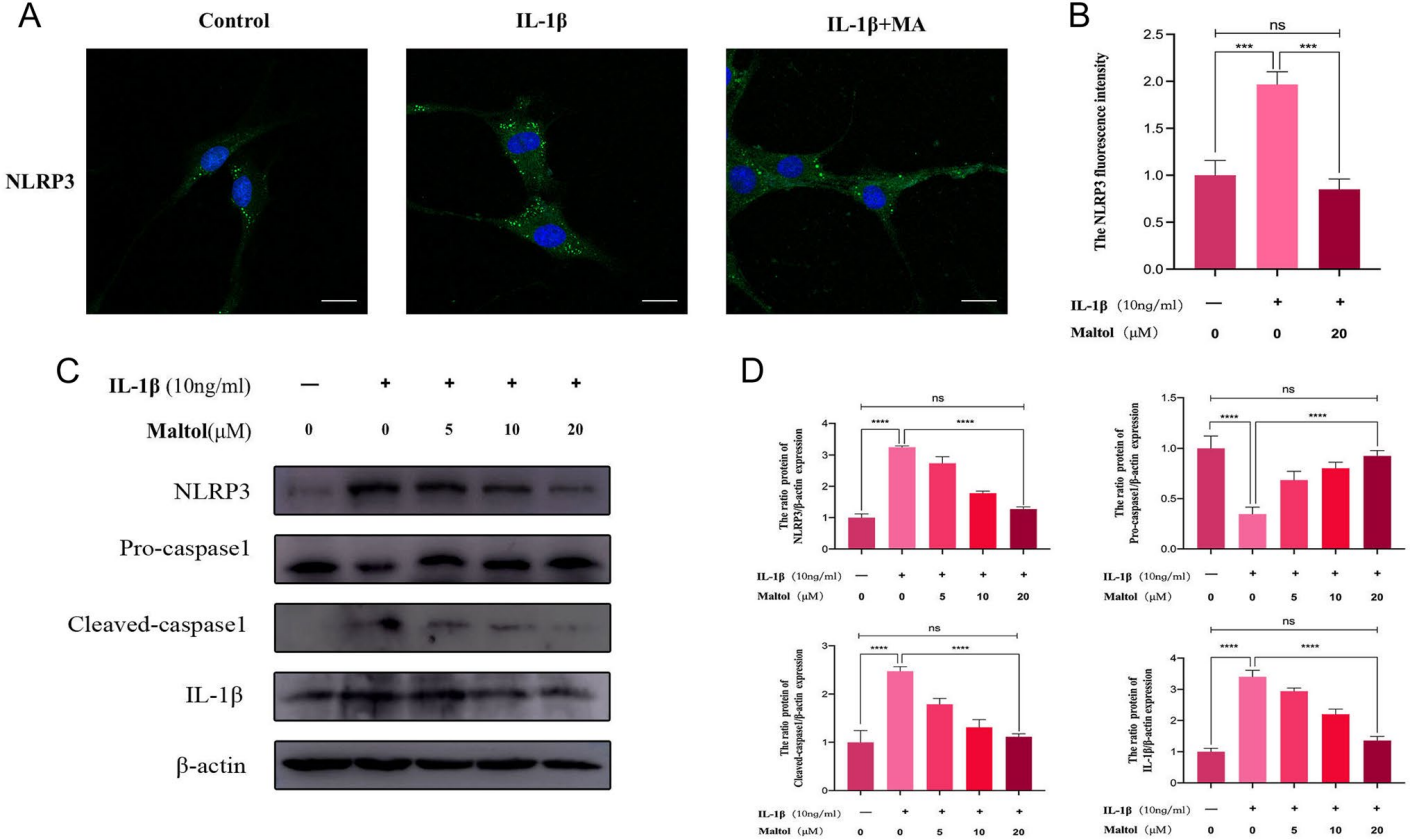
Effects of Maltol on NLRP3 inflammasome-mediated pyroptosis in IL-1β-treated NP cells. **A** NLRP3 was detected by immunofluorescence combined with DAPI staining for nuclei (scale bar: 20 µm). **B** Fluorescence intensity of NLRP3 was determined using ImageJ. **C** Protein expression of NLRP3, Pro-caspasel, Cleaved-caspasel, and IL-1β was detected by Western bolt in NP cells, treated with or without administration of Maltol with IL-1β for 24 h. **D** Levels were determined using ImageJ. The values presented are the means ± SD of three independent experiments. ns *p* > 0.05, *p < 0.05, **p < 0.01, ***p < 0.001, ****p < 0.0001

We noted that NPCs can produce inflammatory factors, including IL-18 and IL-1β, in the presence of IL-1β. According to previous studies, the secretion of IL-18 and IL-1β inflammatory factors by cells is frequently associated with cellular pyroptosis. Therefore, we hypothesized that MA potentially inhibits cellular pyroptosis. The immunofluorescence results showed that NLRP3-inflammasome expression levels in the MA-treated group were closer to those in the control group than to those in the IL-1β group (Fig. 4A, B). Western blot results, the NLRP3/β-actin, Pro-caspase1/β-actin, Cleaved-caspase1/β-actin, and Cleaved-IL-1β/β-actin were significantly elevated in IL-1β-treated NPCs compared with controls, whereas after MA treatment, these ratios were significantly reduced and in a concentration-dependent manner (Fig. 4C, D).

### Effects of MA on the PI3K/AKT/NF-κB signaling pathway in IL-1β-treated NPCs

**Fig. 5:**
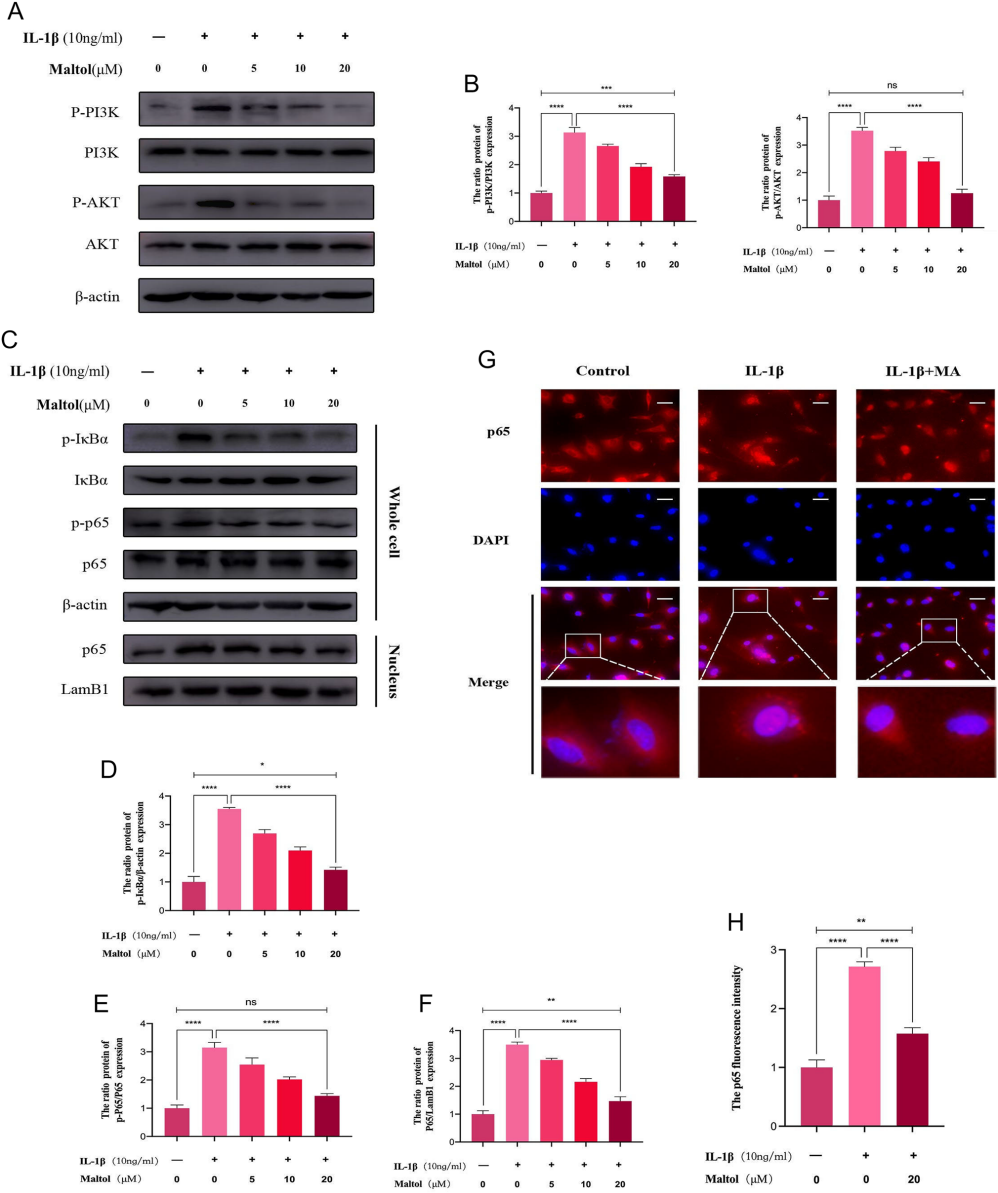
Effects of Maltol on PI3K/AKT/NF-κB signaling pathway activation in IL-1-treated NP cells. **A** Protein expression of the phosphorylation of PI3K, and AKT and their total proteins was detected by Western blot in NP cells, treated with or without administration of Maltol with IL-1β for 24 h. **B** Levels were determined using ImageJ. **C** Protein expression of the phosphorylation of IκBα, and p65, the degeneration of total IκBα, and the and nucleus p65 were detected by Western blot in NP cells, treated with or without administration of Maltol with IL-1β for 24 h. **D**–**F** Levels were determined using ImageJ. **G** p65 was detected by immunofluorescence combined with DAPI staining for nuclei (scale bar: 100 µm). **H** Fluorescence intensity of p65 was determined using ImageJ. The values presented are the means ± SD of three independent experiments. ns *p* > 0.05, *p < 0.05, **p < 0.01, ***p < 0.001, ****p < 0.0001

To investigate whether the PI3K/AKT/NF-κB signaling pathway is regulated by MA in the IDD, we determined the PI3K/AKT/NF-κB signaling pathway was involved in IL-1β-induced NPCs. In IL-1β-treated NPCs, the ratios of p-PI3K/PI3K and p-AKT/AKT were considerably higher than in controls, indicating that the PI3K/AKT signaling pathway was activated. When MA-treated and IL-1β-treated NPCs were compared, the ratios of p-PI3K/PI3K and p-AKT/AKT were considerably lower in the MA-treated NPCs (Fig. 5A, B). As shown in Fig. 5C, IL-1β-induced p65 translocation from the cytoplasm to the nucleus, resulting in promoting the protein expression of phosphorylation of IκBα and P65 and degradated IκBα. MA, by contrast, effectively suppressed the aforesaid effects in a dose-dependent manner (Fig. 5D–F). Furthermore, immunofluorescence images of p65 staining revealed that MA reduced IL-1β-induced p65 nuclear translocation (Fig. 5G, H).

### Molecular docking between MA and PI3K

**Fig. 6:**
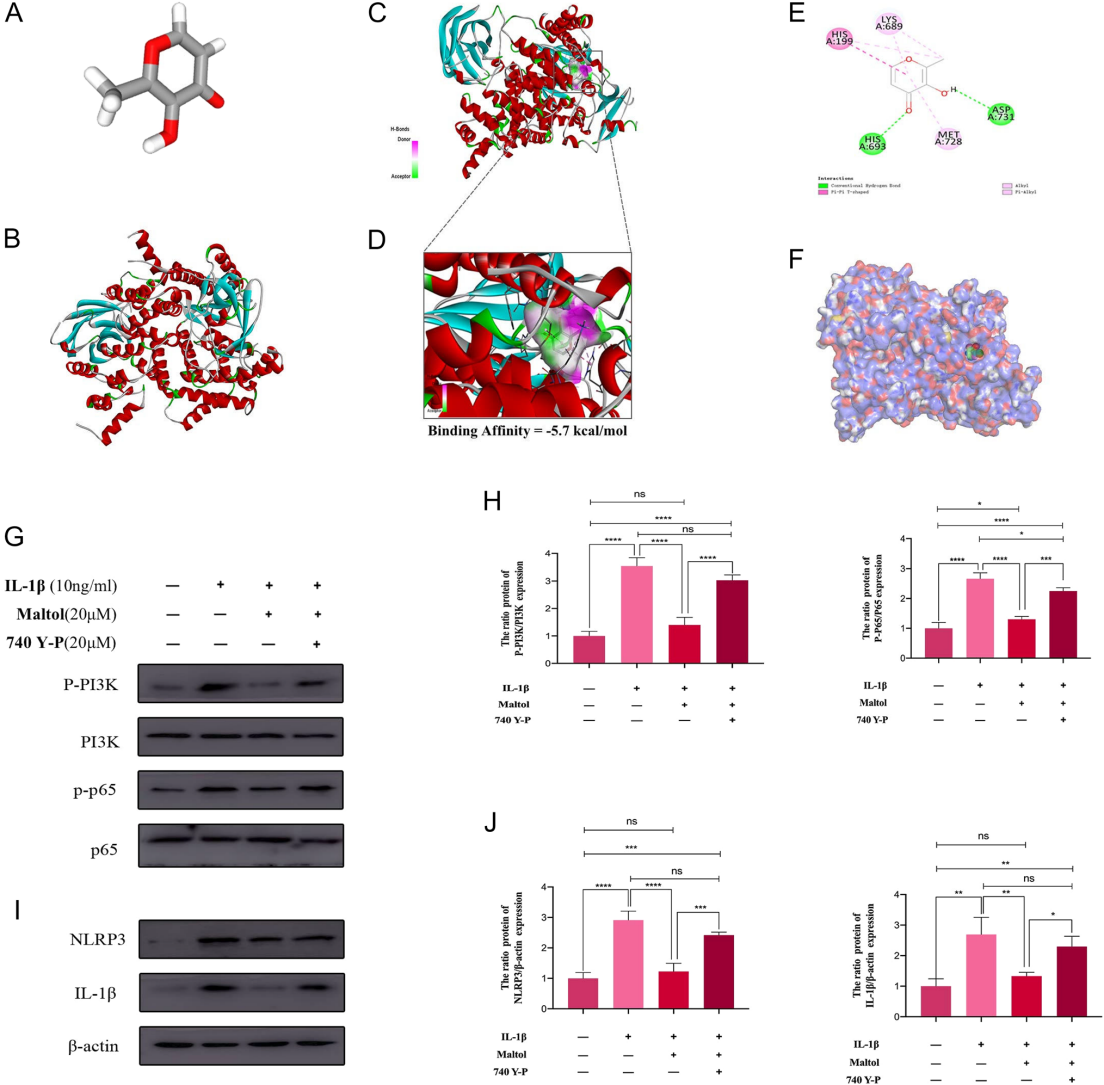
Molecular docking between MA and PI3K. **A** Model of Maltol. **B** Ribbon model of the PI3K. **C**, **D** 2D binding model between Maltol and PI3K and the reaction between Maltol and PI3K; Binding Affmity is -5.7 kcaVmol. **E** Molecules of PI3K that interact with Maltol through Alkyl, hydrogen bonds, Pi–Pi T-shaped and Pi-Alkyl. **F** 3D binding model between Maltol and PI3K. **G** Protein expression of the phosphorylation of PI3K, and p65 and their total proteins was detected by Western blot in NP cells, treated with or without administration of Maltol with IL-1β for 24 h. **H** Levels were determined using ImageJ. **I** Protein expression of NLRP3 and IL-1β was detected by Western blot in NP cells, treated with or without administration of Maltol with IL-1β for 24 h. **J** Values presented are the means ± SD of three independent experiments. ns *p* > 0.05, *p < 0.05, **p < 0.01, ***p < 0.001, ****p < 0.0001

MA and PI3K interaction was analyzed by molecular docking. Several interesting interactions between MA and PI3K were found by examining all of the models. Figure 6A, B illustrates the architectures of MA and PI3K, respectively. According to the docking structure with PI3K, MA and PI3K had high interaction energy (−5.7 kcal/mol) (Fig. 6C, D). MA was identified as molecules interacting with PI3K through alkyl, hydrogen bonds, Pi-Pi T-shaped, and Pi-Alkyl in the two-dimensional binding model (Fig. 6E), and Fig. 6F shows the 3D binding model between MA and PI3K. Next, we examined the effects of MA and PI3K activator 740Y-P in the PI3K/AKT pathway cascaded with the NF-κB pathway and NLRP3 inflammasome-meditated proptosis in IL-1β-induced NPCs by western blot. Consistent with the above described, the ratios of p-PI3K/PI3K, p-p65/p65, NLRP3/β-actin, and IL-1β/β-actin were significantly increased in the IL-1β group, and MA could effectively reduce the ratios of these proteins’ ratios (Fig. 6G, H). However, the ratios of p-PI3K/PI3K (Fig. 6H), p-p65/p65 (Fig. 6H), NLRP3/β-actin (Fig. 6J) and IL-1β/β-actin (Fig. 6J) in 740Y-P + MA group were higher than those in the MA group and were close to those in the IL-1β group (Fig. 6I). The rescue effect of MA was diminished in IL-1β-treated NPCs supplemented with 740Y-P.

### MA ameliorates the IDD process in the mouse model

**Fig. 7:**
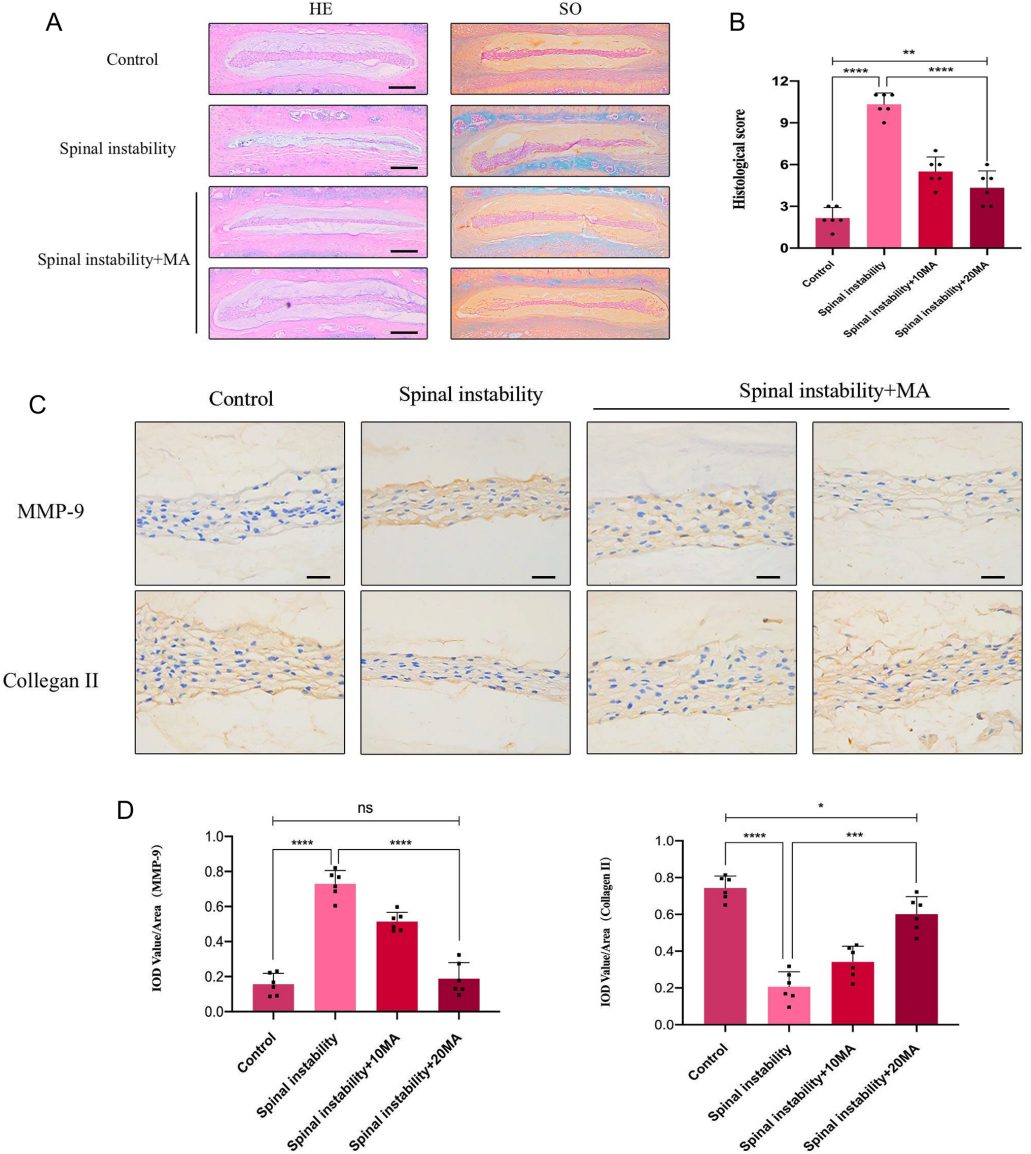
Effect of Maltol on the progression of IDD in the spinal instability mice. **A** Hematoxylin and eosin staining and Farcino O-fast green staining to show intervertebral disc (IVD) status in each group at 12 weeks after surgery in the model of spinal instability (scale bar: 500 µm). **B** IVD histological score of mice in three groups 12 weeks after operation **C** Immunohistochemical staining of MMP-9, Collagen II expression (scale bar: 100 µm). **D**–**G** Their IOD value/area statistics in IVDs at 12 weeks after surgery in the groups. The values presented are the means ± SD of six independent experiments. ns *p* > 0.05, *p < 0.05, **p < 0.01, ***p < 0.001, ****p < 0.0001

**Fig. 8:**
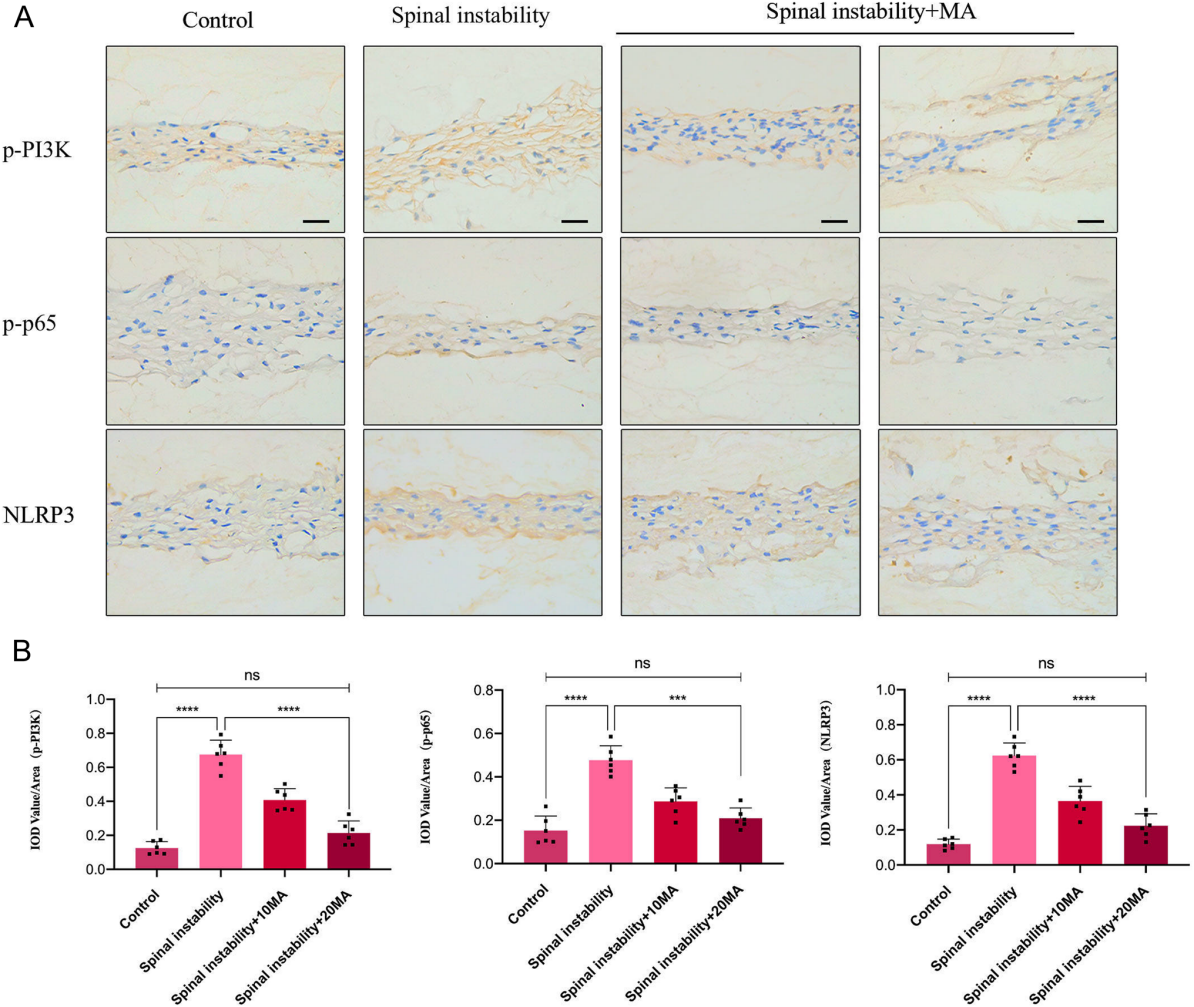
Effect of Maltol on the progression of IDD in the spinal instability mice. **A** Immunohistochemical staining of *p*-PI3K, p-p65, and NLRP3 expression (scale bar: 100 µm). **D**–**G** Their IOD value/area statistics in IVDs at 12 weeks after surgery in the groups. The values presented are the means ± SD of six independent experiments. ns *p* > 0.05, *p < 0.05, **p < 0.01, ***p < 0.001, ****p < 0.0001

To explore the in vivo protective effect of MA on IDD, we first established a surgically induced lumbar instability model in mice. Subsequently, PBS or MA was gavaged twice times a week for 12 weeks, and the lumbar spine tissues of the experimental mice were taken after completing the treatment. HE staining showed that MA improved the disorganization and fragmentation of the laminae (Fig. 7A), while SO staining showed that MA improved the loss of a large number of proteoglycans and glycosaminoglycans (Fig. 7A). The histological score of IVD treated with MA was lower than the spinal instability group (Fig. 7B). Immunohistochemical staining showed that the MMP-9 level increased in the spinal instability group, while MA inhibited the MMP-9 level in the spinal instability + MA group (Fig. 7C, D), also, the Collagen II level was decreased in the spinal instability group, while the Collagen II level was increased in the MA group (Fig. 7C, D). In addition, immunohistochemical staining showed the levels of p-PI3K, p-p65, and NLRP3 of the MA group were similar to control but lower than spinal instability group (Fig. 8A, B).

## Discussion

According to the present study findings, IDD was a major cause of LBP (Smith et al. 2011, Katharina Trompeter et al. 2017). However, the first-line therapy of IDD remains restricted to conservative treatment approaches due to a lack of successful treatment agents (Katharina Trompeter et al. 2017). Research in the past has proved that MA has a positive effect on the treatment of some diseases (Stallmach et al. 2015; Thompson et al. 2009). For example, MA restores ECM balance by inhibiting PI3K/AKT pathway and inhibits osteoarthritis progression (Lu et al. 2021), and inhibits the progression of diabetic nephropathy by regulating NF-κB signaling pathway (Kang et al. 2008). However, the effect of MA on IDD remains to be further studied. As a result of the current investigation, we found that MA ameliorated IDD development through the PI3K/AKT/NF-κB signaling pathway and the NLRP3 inflammasome-mediated pyroptosis pathway, indicating that MA was a viable medication for IDD treatment.

We initially identified a safe dose range of MA in NPCs and used these dosages as the in vitro model concentrations to examine its protective effect. Subsequently, we showed that MA had a cytoprotective impact, reducing IL-1β-induced cytotoxicity. At the same time, we confirmed that MA inhibited excessive catabolism (MMP-3, MMP-9, ADAMTS-4, and ADAMTS-5) and anabolic (collagen II and aggrecan) insufficiency of the ECM, thereby maintaining disc elasticity to absorb compression stresses. Moreover, the effect of MA on ECM proteins was based on the regulation of mRNA transcription as shown by qRT-PCR. Similar to our results, Hongwei Lu et al. found that MA markedly decreased the ADAMTS-5 and MMP-13 protein levels, whose functions were similar to those of ADAMTS-4 and MMP-3, respectively. Therefore, it is not difficult to find that MA can restore the balance between IL-1β-stimulated anabolism and catabolism of NP ECM.

Our study also found that MA decreased the production of pro-inflammatory factors, such as iNOS and COX-2 in NPCs at the mRNA and protein levels. These results implied that MA reduced the expression of pro-inflammatory factors. We also demonstrated that the NF-κB pathway played an essential role in IL-1β-induced inflammation. MA blocked the NF-κB signaling pathway by inhibiting the degradation of Iκb, the phosphorylation of Iκb and the phosphorylation of p65, and inhibit the translocation of p65 to the nucleus. It strongly reduced the NF-κB signaling pathway in the treatment of hepatitis and diabetes (Thompson et al. 2009; Wang et al. 2019), which was similar to our findings. The NLRP3 inflammasome-mediated pyroptosis was found to cause significant cellular production of the inflammatory factors IL-1β and IL-18 (Bakhshi et al. 2022). Furthermore, we found that MA influenced IL-1β-mediated NPC production of inflammatory factors such as IL-1β and IL-18, thus we hypothesized that MA had an inhibitory effect on IL-1β-mediated pyroptosis of NPCs. In a follow-up study, we noted that MA inhibited the formation of NLRP3 inflammasome, resulting in the failure of pro-caspase1 to be cleaved into active cleaved-caspase1, which resulted in the reduction of cellular secretion of inflammatory factors such as IL-1β and IL-18.

Moreover, We found that the PI3K/AKT signaling pathway was implicated in the role of IL-1β-induced NPCs, and MA inhibited the PI3K/AKT signaling pathway by lowering the p-PI3K/PI3K and p-AKTkt/AKT ratios. We discovered that MA interacted with amino acid residues to directly occupy the inhibitory binding pocket of PI3K. We also investigated the effect of PI3K/AKT signaling pathway on MA treatment by adding 740Y-P, a classic PI3K activator, to the MA treatment group. The results showed that 740Y-P attenuated the inhibitory effect of MA on PI3K phosphorylation. Moreover, 740Y-P also attenuated the downstream AKT/NF-κB signaling pathway and NLRP3 inflammasome-mediated pyroptosis. This implies that MA inhibits IDD progression through NF-κB pathway and pyroptosis by PI3K/AKT pathway. In addition, according to Chen F, Jiang G, Liu H, et al., after silencing the key target of NF-κB pathway p65 in NPCs, NLRP3 is significantly inhibited. Therefore, it is also speculated that NLRP3 mediated pyroptosis in the nucleus pulposus cells can also be used as the downstream pathway of the PI3K/AKT/NF-κB/cascade. In addition, In addition, MA may block NLRP3-mediated pyroptosis of nucleus pulposus cells by inhibiting the PI3K/AKT/NF-κB pathway.

We demonstrated that MA had a role not only in vitro but also in surgically induced lumbar instability models in mice. Surgical excision of the spinous process-induced disc degeneration model in mice is widely used to simulate IDD. We found that MA improved tissue disorganization and fragmentation of the laminae, improved loss of anabolic proteins (Collagen II), and inhibited intra-disc inflammation and pyroptosis (MMP-9, p-PI3K, p-p65, and NLRP3), suggesting a protective effect of MA on the intra-disc to disc in mice.

## Conclusions

**Fig. 9:**
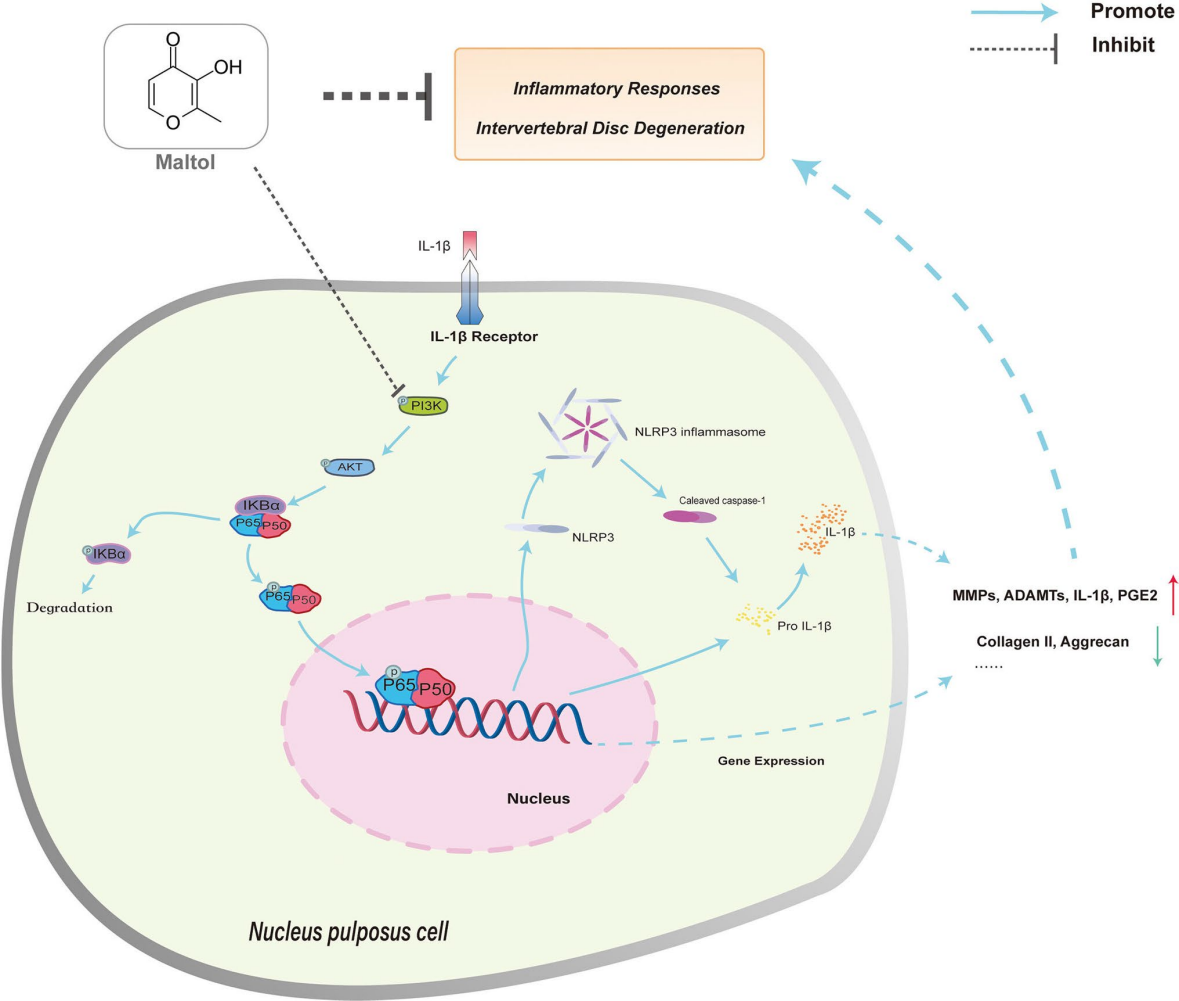
Model proposing the role of maltol in intervertebral disc degeneration therapy

Our data indicated that MA alleviates intra-and peri-disc matrix degradation and intervertebral disc inflammation (Fig. 9) by inhibiting PI3K/AKT/NF-κB signaling and NLRP3 inflammasome-mediated pyroptosis, thereby maintaining intra- and peri-disc microenvironment homeostatic and moderating intervertebral disc degeneration. These observations identified MA as a potential treatment for intervertebral disc degeneration.

## Data Availability

The raw data supporting the conclusions of this article will be made available by the authors, without undue reservation, to any qualifed researcher.

## Abbreviations

ADAMTS: A disintegrin and metalloprotease with thrombospondin
DMSO: Dimethyl sulfoxide
DAPI: 4ʹ,6-Diamidino-2-phenylindole hydrochloride
ECM: Extracellular matrix
EdU: 5-Ethynyl-2′-deoxyuridine
HE: Hematoxylin–eosin
IVD: Intervertebral disc
MA: Maltol
MMP: Motifs matrix metalloproteinase
NP: Nucleus pulposus
PGE2: Prostaglandin E2
SO: SafraninO-fast green
TNF-α: Tumor necrosis factor-α
740Y-P: 740YPDGFR
